# Efficacy of PD-1 inhibitor with neoadjuvant chemotherapy in hypopharyngeal and oropharyngeal cancer

**DOI:** 10.3389/fonc.2024.1450830

**Published:** 2024-09-24

**Authors:** Xue-Ying Liu, Han-jing Shang-guan, Wei Zhang, Shuai Chen, Xian-Yang Luo

**Affiliations:** ^1^ Department of Otolaryngology Head and Neck Surgery, The First Affiliated Hospital of Xiamen University, School of Medicine, Xiamen University, Xiamen, China; ^2^ State Key Laboratory of Vaccines for Infectious Diseases, Xiang’an Biomedicine Laboratory, State Key Laboratory of Molecular Vaccinology and Molecular Diagnostics, National Innovation Platform for Industry-Education Integration in Vaccine Research, School of Public Health, Xiamen University, Xiamen, China; ^3^ Department of Otolaryngology Head and Neck Surgery, Xiamen Key Laboratory of Otolaryngology Head and Neck Surgery, Xiamen, Fujian, China

**Keywords:** PD-1 inhibitor, oropharyngeal cancer, hypopharyngeal cancer, laryngeal preservation, neoadjuvant chemotherapy

## Abstract

**Objective:**

This study is aimed to evaluate the efficacy and safety of PD-1 inhibitors combined with neoadjuvant chemotherapy in patients with locally advanced hypopharyngeal and oropharyngeal cancer prior to surgical resection.

**Methods:**

This retrospective analysis included 42 patients diagnosed with locally advanced hypopharyngeal and oropharyngeal cancer. The efficacy, safety, survival, and laryngeal preservation rate were evaluated.

**Results:**

A total of 42 patients were included in this retrospective analysis, of whom 28 had hypopharyngeal cancer and 14 had oropharyngeal cancer. Of the 42 patients, 14 (33.3%) achieved a pathological complete response (PCR) at the primary site, 20 (47.6%) achieved a major pathological response (MPR), and 8 (19%) had an incomplete pathological response (IPR) at the primary lesion. A PCR at both the primary site and the neck lymph nodes was observed in 9 patients (21.4%). The laryngeal preservation rate was 92.9% (26/28) in patients with hypopharyngeal cancer. The median follow-up time was 10.5 months. The median progression-free survival (PFS) was 26.42 months (95% CI, 23.416–29.424), and the median overall survival (OS) was 27.1 months (95% CI, 24.316–29.884). The 1-year PFS rate was 83.1%, and the 1-year OS rate was 85.9%.

**Conclusion:**

Combination therapy with PD-1 inhibitors and neoadjuvant chemotherapy has demonstrated superior efficacy and safety as a preoperative treatment for locally advanced hypopharyngeal and oropharyngeal cancer. Notably, this treatment regimen does not increase the risk of severe postoperative complications and has shown promising results in improving laryngeal preservation rates.

## 1 Introduction

Hypopharyngeal carcinoma (HPC) is a rare malignancy among head and neck squamous cell carcinomas and carries a dismal prognosis, with a 5-year overall survival (OS) rate of only approximately 30%–35% (1, 2). HPC is often asymptomatic in its early stages, leading to a high proportion of patients presenting with advanced-stage disease at diagnosis (3). However, the standardized treatment protocol for patients with locally advanced hypopharyngeal carcinoma (LAHPC) has yet to be established. Although laryngeal preservation as a clinical paramount concern has achieved a significantly higher laryngeal preservation rate under the treatment of chemoradiotherapy (CRT) or induction chemotherapy followed by chemoradiotherapy (ICT), the overall survival (OS) probability is not improved (4). The incidence of HPV-associated oropharyngeal squamous cell carcinoma (OPSCC) has witnessed a steady increase in recent years (5–7). Meanwhile, patients with locally advanced oropharyngeal cancer, the primary tumor is frequently too large to be resected in its entirety (8).

Head and neck squamous cell carcinoma (HNSCC) exhibits a unique tumor microenvironment characterized by immune escape mechanisms, including the hijacking of immune cells by HNSCC cells. Programmed Death-1 (PD-1) is an immune checkpoint molecule that inhibits T cell activity upon binding to programmed cell death-ligand 1 (PD-L1). Tumor cells can evade immune surveillance by upregulating PD-L1 expression. Immune checkpoint inhibitors (ICIs) function by blocking the PD-1/PD-L1 interaction, thereby restoring T cell-mediated antitumor immunity and suppressing tumor growth (9, 10). ICIs have emerged as a rapidly evolving therapeutic strategy in the management of head and neck malignancies. Clinical trials have demonstrated the efficacy and safety of PD-1 inhibitors in patients with HNSCC (11, 12). Recent studies have also evaluated the efficacy and safety of neoadjuvant chemotherapy (NAC) in combination with ICIs in patients with HNSCC (13, 14).

Due to limited research on the combination of PD-1 inhibitors with NAC in locally advanced hypopharyngeal and oropharyngeal squamous cell carcinoma, this study aimed to assess the efficacy and safety of PD-1 inhibitor plus NAC as a preoperative treatment for these cancers.

## 2 Materials and methods

### 2.1 Subjects

This retrospective study enrolled patients diagnosed with locally advanced hypopharyngeal or oropharyngeal squamous cell carcinoma at the First Affiliated Hospital of Xiamen University between February 2021 and September 2023. Inclusion criteria: (1) Histologically or cytologically confirmed diagnosis of squamous cell carcinoma; (2) Patients with locally advanced (stage III-IV) hypopharyngeal or oropharyngeal squamous cell carcinoma; (3) Received at least two cycles of ICIs combined with neoadjuvant chemotherapy; (4) Underwent surgical treatment following ICIs combined with neoadjuvant chemotherapy. Exclusion criteria: (1) Patients with recurrent and metastatic hypopharyngeal and oropharyngeal carcinoma; (2) Allergic to any of the drugs used in this study; (3) Unable to tolerate general anesthesia surgery. (4) Active pulmonary tuberculosis patients. The study received approval from the Ethics Committee of the First Affiliated Hospital of Xiamen University. All patients provided written informed consent before treatment.

### 2.2 Treatment

Treatment decisions for each patient were made by a multidisciplinary team (MDT) after reviewing pathological results, imaging findings, narrow-band imaging with electronic laryngoscopy, and preoperative staging. During the neoadjuvant therapy period, the patients received ICI (Pembrolizumab 200mg; Tislelizumab 200mg; Nivolumab 240mg; Camrelizumab 200mg) combined with paclitaxel (Albuminbound) 260mg/m^2^ and cisplatin 75mg/m^2^. The drugs were administered on the first day of a three-week treatment cycle.

Subsequently, surgical intervention was performed approximately four weeks after the completion of neoadjuvant therapy. The surgical approach was selected based on the extent of therapeutic response observed following the initial treatment regimen. The surgical intervention encompassed primary tumor resection via transoral cold plasma resection, partial laryngectomy, or total laryngectomy, accompanied by prophylactic or therapeutic neck lymph node dissection (**Table 1**). Postoperatively, all patients received either radiotherapy or concurrent chemoradiotherapy (CCR). The prescribed dose for the tumor bed was 60-66 gray (Gy) delivered over 32 fractions, while the neck received a dose of 54-60Gy/32f. Platinum-based concurrent chemoradiotherapy was administered to patients with extranodal invasion, multiple lymph node metastases, or positive margins.

**Table 1:** Patient's surgical condition.

Surgical procedure	hypopharyngeal cancer	oropharyngeal cancer
TL+NLND	2	0
PL+NLND	3	0
TORS+NLND	23	14

TL, total laryngectomy; PL, partial laryngectomy; TORS, transoral resection of primary lesion; NLND, neck lymph node dissection.

### 2.3 Clinical efficacy assessment

Forty-two patients underwent neck computed tomography (CT) or magnetic resonance imaging (MRI) scans, standard white-light laryngoscopy, and narrow-band imaging (NBI) examinations both before and after neoadjuvant therapy to assess the extent of their lesions. According to the Response Evaluation Criteria in Solid Tumors (RECIST 1.1), patients were evaluated for response to ICIs with NAC using radiologic scans, which included complete response (CR), partial response (PR), progressive disease (PD), and stable disease (SD).

OLYMPUS ENT-VT2 CV170 electronic endoscope system was selected, which offers standard white-light endoscopy and NBI endoscopy. The NBI mode leverages the classification criteria proposed by Ni Xiaoguang to effectively categorize the observed lesions (15). Type I: Typically observed in normal mucosa, vocal polyps, cysts, granulation tissue, and scarred mucosa. Type II: Lesions associated with inflammation. Type III: Frequently observed in leukoplakia, characterized by epithelial hyperplasia and keratinization. Type IV: Lesions exhibiting mild to moderate dysplasia of squamous epithelium. Type Va: Severe dysplasia and carcinoma *in situ* are most commonly observed in Type Va lesions. Type Vb and Vc: Primarily associated with invasive carcinoma, representing more advanced stages of malignant transformation.

The postoperative histology results of the resected primary lesion specimen and/or lymph node tissue were used to assess the treatment response. Complete pathological response (PCR) is defined as the absence of any viable tumor cells in both the resected primary lesion and all associated lymph nodes. Major pathological response (MPR) is defined as the presence of ≤10% viable tumor cells in the resected primary tumor. Incomplete pathological response (IPR) is defined as the presence of 10% or more viable tumor cells in the resected primary lesion.

### 2.4 Adverse reactions

Adverse events were recorded using in-hospital medical records and through telephone follow-up. The events were graded in accordance with the Common Terminology Criteria for Adverse Events (CTCAE v5.0), which classifies adverse events into five levels including G1: mild - mild symptoms that do not require treatment, G2: moderate - moderate symptoms that may require treatment, G3: severe - severe symptoms that require treatment, G4: threat to life- symptoms that are life-threatening and require urgent treatment, and G5: death-adverse events resulting in death. No serious immune-related events appeared in the enrolled cohorts of this study. For grades I-II related adverse reactions, symptomatic treatment is given. In brief, patients with rashes/pruritus were treated with antihistamines or even glucocorticoids. Patients with hepatorenal toxicity were regularly monitored to rule out other causes of hepatorenal toxicity, and were given glucocorticoid therapy if necessary.

### 2.5 Statistical methods

All statistical analyses were conducted using SPSS version 26.0 (IBM Corp., Armonk, NY, USA). Overall survival (OS) was defined as the time from the initiation of neoadjuvant therapy to the date of death from any cause. Progression-free survival (PFS) was defined as the time from the initiation of neoadjuvant therapy to the date of disease progression or death. Median OS and PFS were calculated using the Kaplan–Meier method. Categorical variables were described as frequencies (percentages) and analyzed with the chi-square test or Fisher’s exact test.

## 3 Results

### 3.1 Patient characteristics

**Figure 1:**
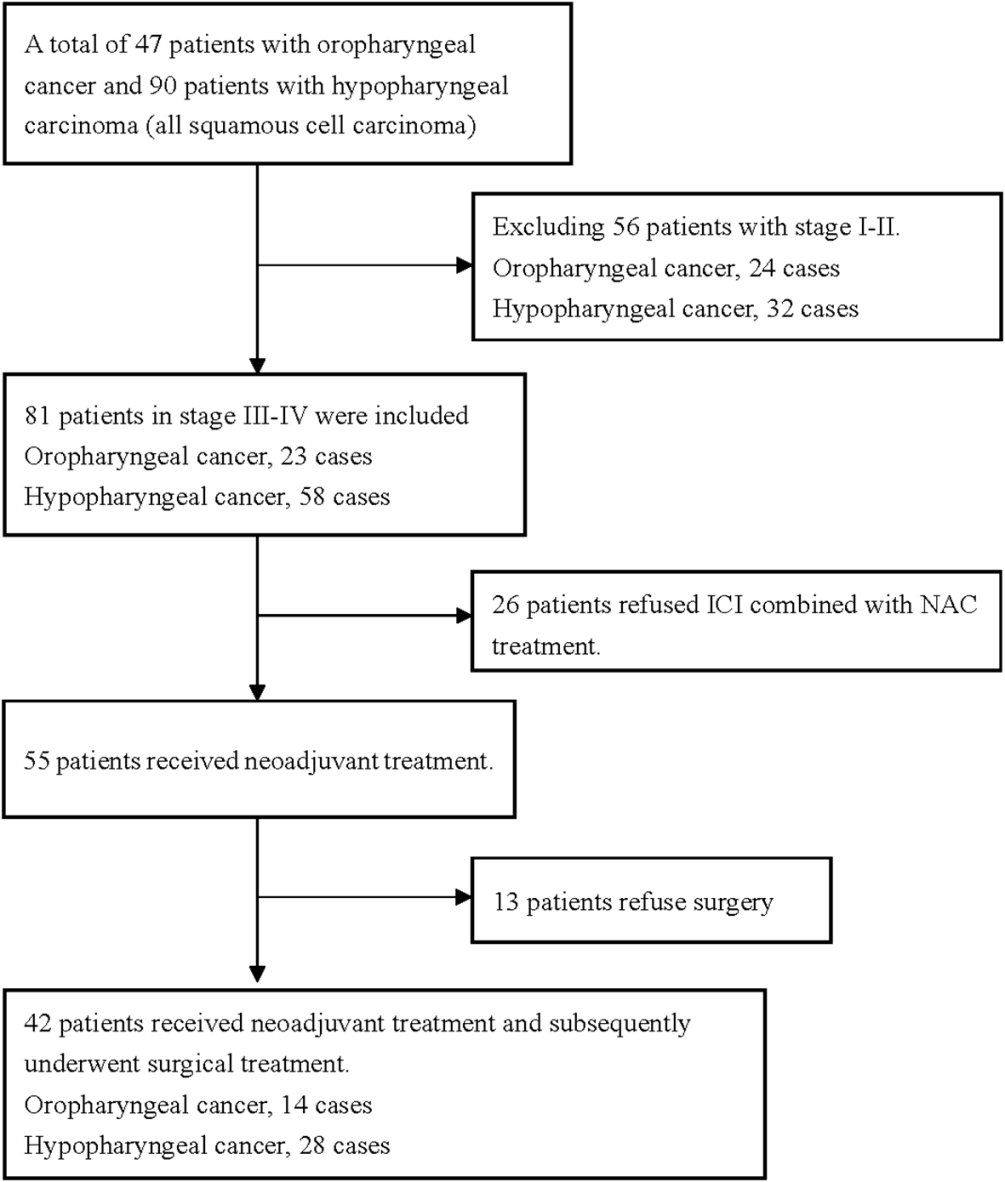
Incorporate the patient flowchart.

A total of 42 patients were enrolled in the study (**Figure 1**). The cohort comprised 39 males and 3 females, with an age range of 39–79 years and a mean age of 59.3 years. The tumor locations were the oropharynx and hypopharynx in 14 (33.3%) and 28 (66.7%) cases, respectively. There were 13 cases of stage III disease and 29 cases of stage IV disease. Additionally, all 42 patients had a pretreatment NBI classification of Va-Vc. All patients received at least two cycles of ICI in combination with NAC. The ICIs used by patients included tislelizumab (n=21), nivolumab (n=3), camrelizumab (n=8), and pembrolizumab (n=10) (**Table 2**).

**Table 2:** Basic information of patients.

Characteristic	patients(N=42)	%
Average age	59.3 ± 7.65	
Sex		
Male	39	92.86%
Female	3	7.14%
Smoking		
Yes	32	76.19%
No	10	23.81%
Drinking		
Yes	28	66.67%
No	14	33.33%
T- stage		
T1	1	2.38%
T2	13	30.95%
T3	18	42.86%
T4	10	23.81%
N -stage		
N0	4	9.52%
N1	8	19.05%
N2	29	69.05%
N3	1	2.38%
TNM-stage		
III	13	30.95%
IV	29	69.05%
Primary site		
oropharynx	14	33.33%
hypopharynx	28	66.67%
CPS		
<1	2	4.76%
1-19	22	52.38%
≥20	18	42.86%
PD-1 inhibitors		
Pembrolizumab	10	23.81%
Tislelizumab	21	50.00%
Nivolumab	3	7.14%
Camrelizumab	8	19.05%
NBI after neoadjuvant therapy		
I-IV	26	61.90%
V	16	38.10%

CPS, combined positive score; T, tumor; N, nodal; M, metastatic; PD-1, programmed death 1; NBI, narrow-band imaging.

Three patients received three cycles of neoadjuvant therapy due to the COVID-19 pandemic, and one patient received three cycles due to personal reasons. Another patient received four cycles due to poor response to neoadjuvant therapy. The remaining 37 patients all received two cycles of neoadjuvant therapy.

### 3.2 Imaging assessment after neoadjuvant therapy

**Figure 2:**
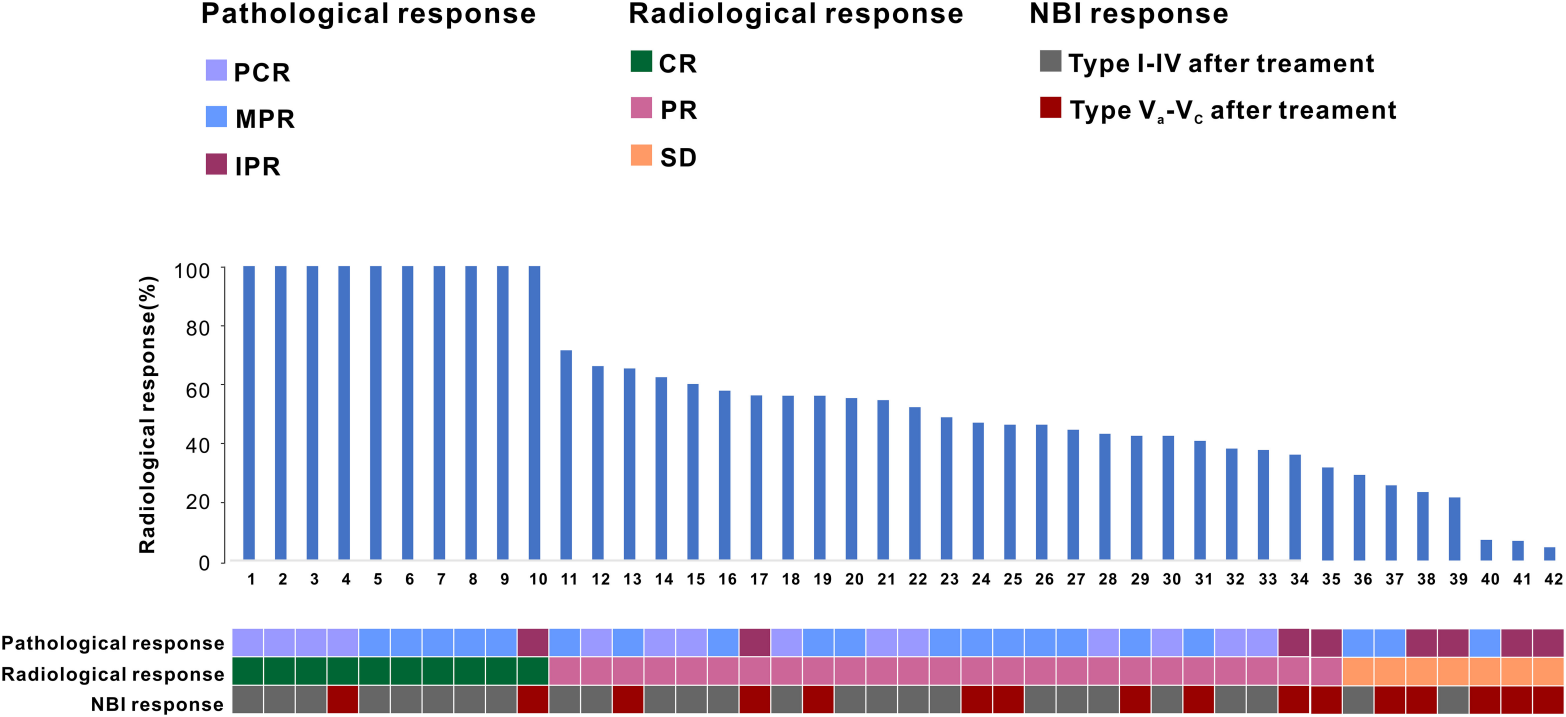
The patient’s treatment response following PD-1 inhibitor combined with neoadjuvant chemotherapy.

Following neoadjuvant therapy, a comprehensive head and neck imaging examination was conducted to assess treatment response. Among the 42 patients, 10 (23.8%) achieved complete radiological remission (CR), including 2 cases of oropharyngeal cancer and 8 cases of hypopharyngeal cancer. Notably, 1 of the 8 patients with hypopharyngeal cancer demonstrated an incomplete pathological response (IPR) on pathological examination. Furthermore, 25 patients (59.5%) exhibited partial remission (PR) based on imaging assessment. Of these, 11 cases were classified as oropharyngeal cancer and 14 cases were classified as hypopharyngeal cancer. Additionally, 7 patients (16.7%) showed stable disease (SD) according to imaging findings, with only 1 case (1/7) involving oropharyngeal cancer. Of note, among the seven SD patients, three cases achieved a major pathological response (MPR) on pathological assessment (**Figure 2**).

### 3.3 Narrow-band imaging of electronic laryngoscope after neoadjuvant therapy

As shown in **Table 3**, among the 26 patients, benign lesions were identified in narrow-band imaging (NBI) endoscopy, with 6 cases of type II, 9 cases of type III, and 11 cases of type IV. There was a total of 16 cases of type V lesions (Va, Vb, Vc), with a concordance rate of 93.75% (15/16) compared to pathological examination. Using type V as the diagnostic criterion for malignant lesions, NBI endoscopy exhibited a sensitivity of 57.69% and a specificity of 93.75% in diagnosing lesions after immunotherapy and chemotherapy.

**Table 3:** Relationship between NBI classification and postoperative pathological results.

	Benign	Mild atypical hyperplasia	Moderate atypical hyperplasia	Severe atypical hyperplasia	Invasive carcinoma	total
I	0	0	0	0	0	0
II	3	0	1	1	1	6
III	4	0	0	1	4	9
IV	4	1	2	1	3	11
Va	0	0	1	0	2	3
Vb	0	0	0	0	9	9
Vc	0	0	0	0	4	4
total	11	1	4	3	23	42

### 3.4 Pathological assessment of response after neoadjuvant therapy

A total of 42 patients underwent surgery approximately 4 weeks following the final ICI+NAC regimen. Among these patients, 5 individuals diagnosed with hypopharyngeal cancer underwent open laryngectomy in combination with neck lymph node dissection. Specifically, 3 patients underwent partial laryngectomy, while the remaining 2 patients underwent total laryngectomy. The remaining patients underwent transoral low-temperature plasma primary lesion resection combined with neck lymph node dissection. The laryngeal preservation rate among patients with hypopharyngeal cancer was 92.9% (26/28). Importantly, all patients achieved complete surgical resection (R0 resection), indicating the successful removal of the primary tumor with negative surgical margins. This was confirmed through postoperative pathological evaluation.

**Figure 3:**
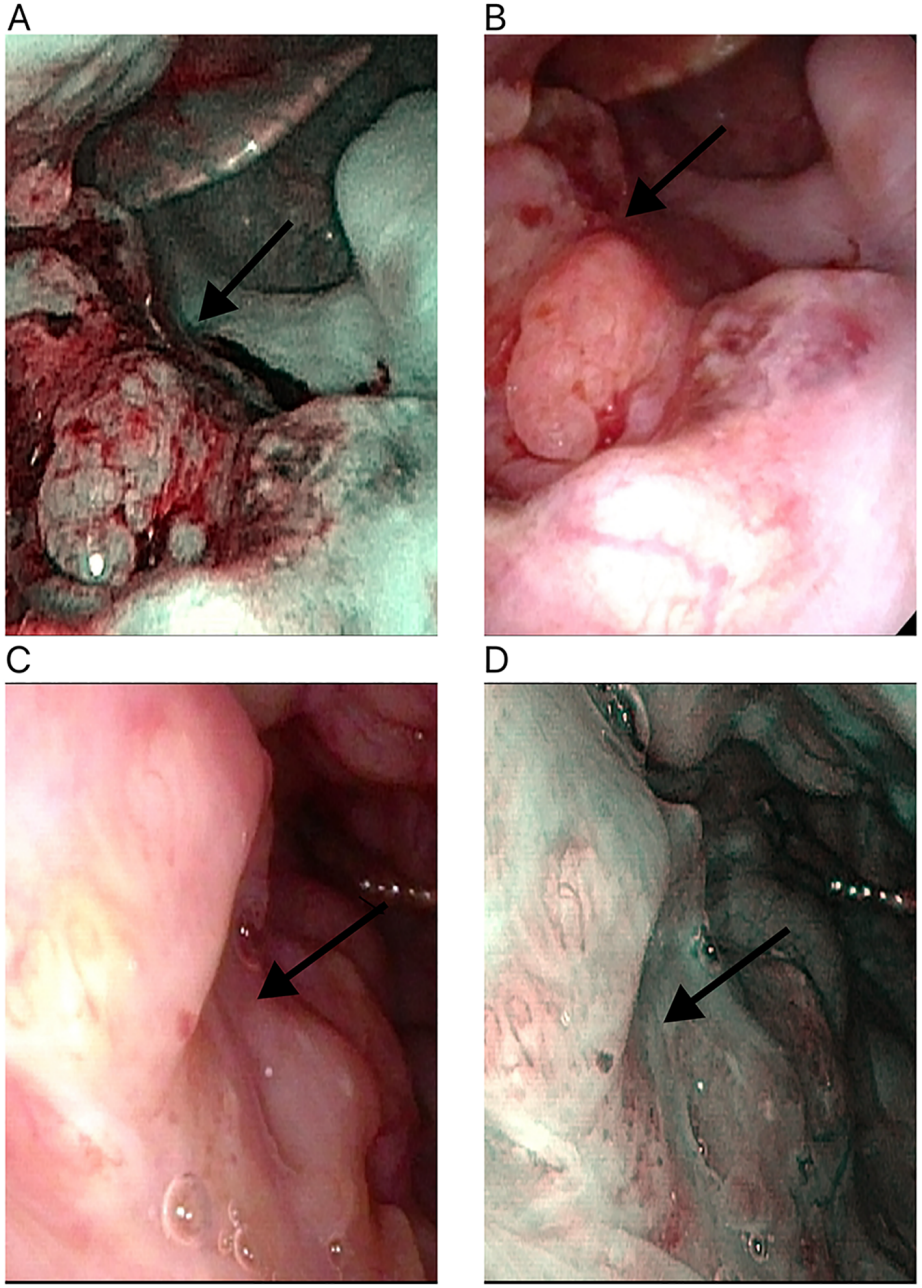
Changes in the primary tumor site in white light laryngoscopy and NBI mode after neoadjuvant therapy for oropharyngeal cancer. **(A)** White light laryngoscopy assessment before neoadjuvant therapy. **(B)** NBI endoscopy assessment before neoadjuvant therapy. **(C)** White light laryngoscopy assessment after neoadjuvant therapy. **(D)** NBI endoscopy assessment after neoadjuvant therapy. NBI, narrow-band imaging. The white arrow points to the primary tumor site.

**Figure 4:**
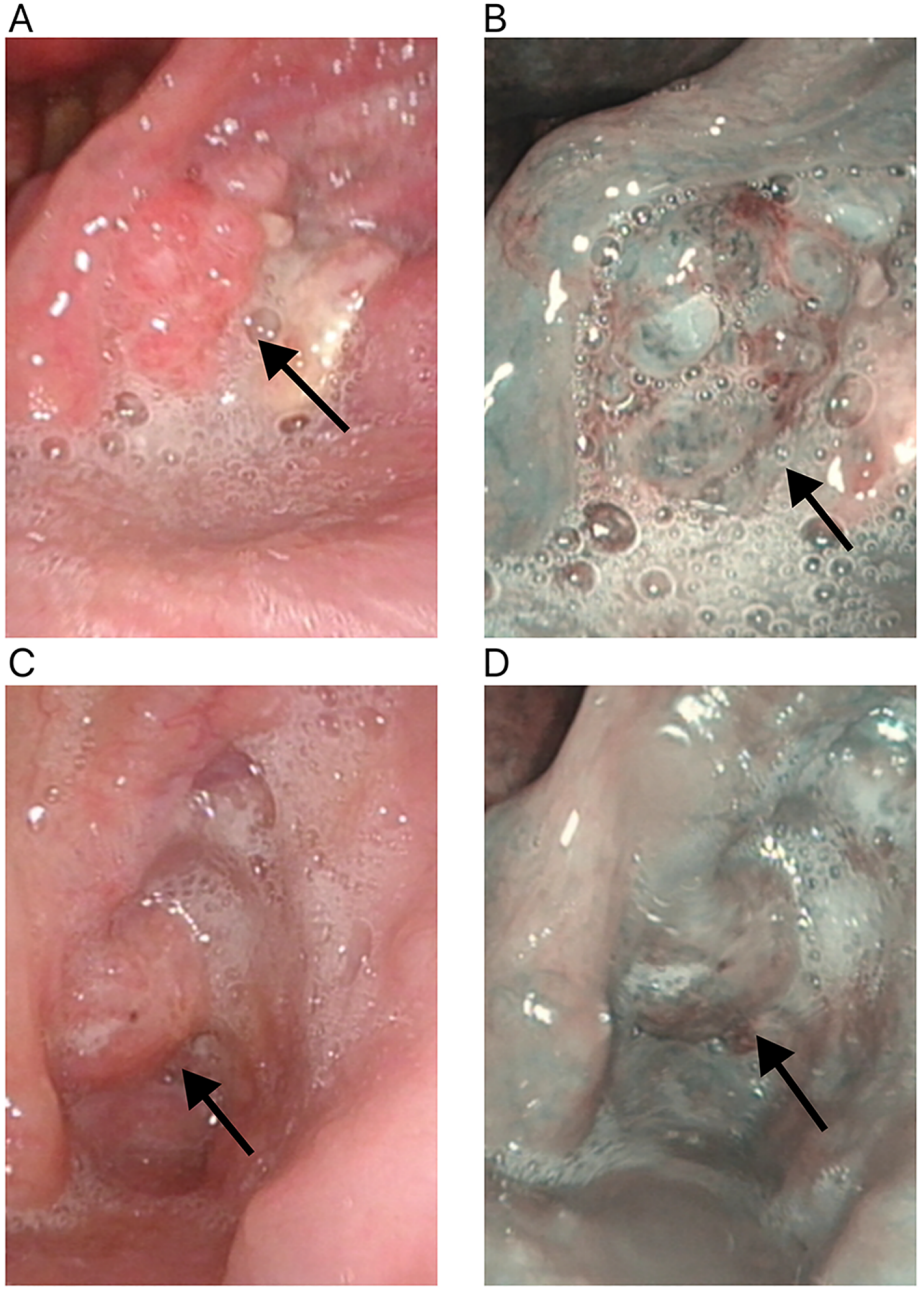
Changes in the primary tumor site in white light laryngoscopy and NBI mode after neoadjuvant therapy for hypopharyngeal cancer. **(A)** White light laryngoscopy assessment before neoadjuvant therapy. **(B)** NBI endoscopy assessment before neoadjuvant therapy. **(C)** White light laryngoscopy assessment after neoadjuvant therapy. **(D)** NBI endoscopy assessment after neoadjuvant therapy. NBI, narrow-band imaging. The white arrow points to the primary tumor site.

In a cohort of 42 patients, 14 patients (33.3%) achieved a pathological complete response (PCR) in their primary lesions. Among these patients,13 patients (92.9%) had NBI classification of type I-IV, while 1 patient (7.1%) had type V (**Figures 3**, **4**). In terms of imaging evaluation, 4 patients (28.6%) achieved a complete response (CR), while 10 patients (71.4%) achieved a partial response (PR) (**Figure 2**).

Among these patients, 20 cases (20/42) demonstrated significant treatment efficacy, with their primary lesions achieving a major pathological response (MPR). Of these 20 cases, 12 cases (12/20) had narrow-band imaging (NBI) types I-IV, while 8 cases (8/20) had NBI type V (**Figures 3**, **4**). In terms of imaging evaluation, 5 patients (5/20) achieved a complete response (CR), 12 patients (12/20) achieved a partial response (PR), and only 3 patients (3/20) were classified as having stable disease (SD) (**Figure 2**).

A subset of 8 cases (19%) exhibited an incomplete pathological response (IPR) to the treatment. Among these 8 patients, the distribution of narrow-band imaging (NBI) types revealed that only 1 case (12.5%) was classified as NBI type IV, while the remaining 7 cases (87.5%) were categorized as NBI type V (**Figures 3**, **4**). Regarding the assessment of treatment response through imaging evaluation, among these 8 IPR patients, only 1 patient (12.5%) achieved a complete response (CR), 3 patients (37.5%) achieved a partial response (PR), while 4 patients (50%) were classified as having stable disease (SD). The overall efficacy rate was 81% (34/42) (**Figure 2**).

The postoperative pathology of cervical lymph nodes in 15 patients reached PCR (35.7%). Nine patients achieved PCR (21.4%) in both the primary tumor and the neck lymph nodes. Furthermore, we found that significant downgrading on NBI assessment was associated with a higher probability of PCR or MPR (73.53%% vs. 12.5%, P=0.003) (**Table 4**).

**Table 4:** Pathological characteristics of the patient.

Variable	PCR/MPR	IPR	*P*
**N**	34	8	
**Gender**			0.479
Male	32 (94.12%)	7 (87.50%)	
Female	2 (5.88%)	1 (12.50%)	
**Smoking**			0.369
No	7 (20.59%)	3 (37.50%)	
Yes	27 (79.41%)	5 (62.50%)	
**Drinking**			0.092
No	9 (26.47%)	5 (62.50%)	
Yes	25 (73.53%)	3 (37.50%)	
**Primary site**			0.697
Oropharynx	12 (35.29%)	2 (25.00%)	
Hypopharynx	22 (64.71%)	6 (75.00%)	
**T- stage**			0.528
T1	1 (2.94%)	0 (0.00%)	
T2	12 (35.29%)	1 (12.50%)	
T3	14 (41.18%)	4 (50.00%)	
T4	7 (20.59%)	3 (37.50%)	
**N -stage**			0.653
N0	3 (8.82%)	1 (12.50%)	
N1	7 (20.59%)	1 (12.50%)	
N2	23 (67.65%)	5 (62.50%)	
N3	1 (2.94%)	1 (12.50%)	
**TNM-stage**			> 0.999
III	11 (32.35%)	2 (25.00%)	
IV	23 (67.65%)	6 (75.00%)	
**NBI after neoadjuvant therapy**			0.003**
I-IV	25 (73.53%)	1 (12.50%)	
V	9 (26.47%)	7 (87.50%)	
**CPS**			0.81
<1	2 (5.88%)	0 (0.00%)	
1-19	17 (50.00%)	5 (62.50%)	
≥20	15 (44.12%)	3 (37.50%)	

CPS, combined positive score; T, tumor; N, nodal; M, metastatic; PD-1, programmed death 1; PCR, pathological complete response; MPR, major pathological response; IPR, incomplete pathological response; NBI, narrow-band imaging. **, P < 0.01.

### 3.5 Safety assessment

Based on the recorded safety assessment, no grade 3–5 adverse events were observed in any of the 42 patients. The most frequent grade 1–2 adverse events were alopecia (64.3%), rash or pruritus (59.5%), fatigue (42.9%), and nausea or vomiting (35.7%). For grades 1-2 related adverse reactions, symptomatic treatment is given. Patients with rash/itchy skin are treated with antihistamines, and if necessary, corticosteroids are administered accordingly. Patients exhibiting hepatotoxicity or nephrotoxicity should have relevant parameters monitored regularly to rule out other causes of liver or kidney toxicity. If needed, corticosteroid therapy is initiated. We routinely used polyethylene glycol recombinant human granulocyte colony-stimulating factor for patients undergoing chemotherapy, thus there were no patients who had grade 3-5 myelosuppression (**Table 5**).

**Table 5:** The relevant adverse reactions of PD-1 inhibitors combined with neoadjuvant chemotherapy.

Adverse reactions	Grade 1-2 (%)	Grade 3	Grade 4	Grade 5
Leukopenia	5 (11.9)	0	0	0
Hypokalemia	3 (7.1)	0	0	0
Hypocalcemia	2 (4.8)	0	0	0
Anemia	8 (19)	0	0	0
Rash/ pruritus	25 (59.5)	0	0	0
Fatigue	18 (42.9)	0	0	0
Nausea/Vomiting	15 (35.7)	0	0	0
Diarrhea	3 (7.1)	0	0	0
Thyroid dysfunction	6 (14.3)	0	0	0
Alopecia	27 (64.3)	0	0	0
Hepatotoxicity	5 (11.9)	0	0	0
Nephrotoxicity	2 (4.8)	0	0	0

### 3.6 Survival

**Figure 5:**
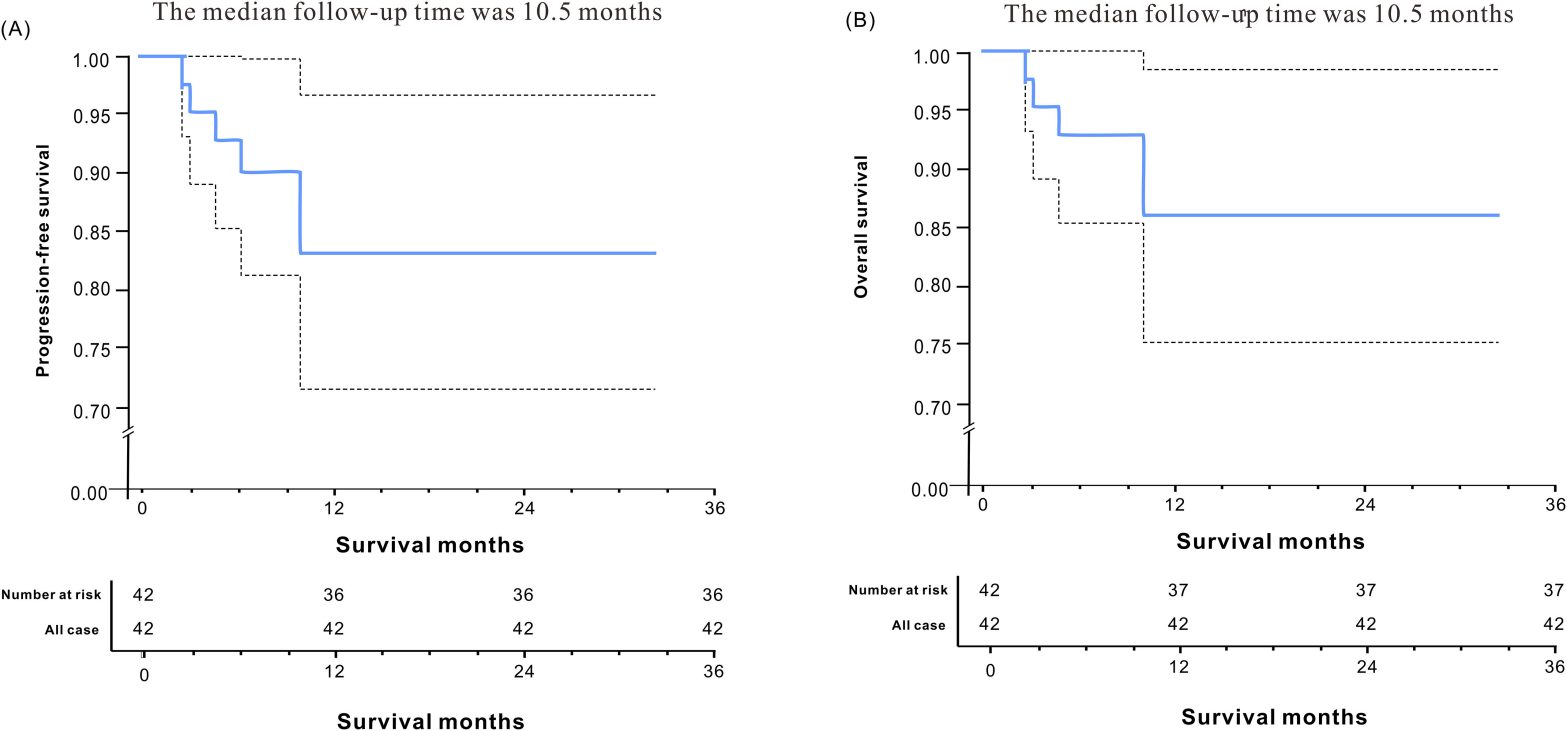
progression-free survival **(A)** and Overall survival **(B)** of the enrolled patients.

The median follow-up time was 10.5 months. The median progression-free survival (PFS) was 26.42 months (95% CI, 23.416-29.424), and the median overall survival (OS) was 27.1 months (95% CI, 24.316-29.884). The 1-year progression-free survival rate was 83.1%, and the 1-year overall survival rate was 85.9% (**Figure 5**).

## 4 Discussion

This study investigated the efficacy and laryngeal preservation of neoadjuvant chemotherapy combined with immunotherapy in patients with locally advanced hypopharyngeal and oropharyngeal carcinoma. The survival and laryngeal preservation outcomes for oropharyngeal and hypopharyngeal carcinoma patients in our study were promising, with a 1-year progression-free survival rate of 83.1%, a 1-year overall survival rate of 85.9%, and a laryngeal preservation rate of 92.9%. These findings suggest that this treatment approach has significant clinical implications. The interaction between PD-1 with PD-L1 facilitates the evasion of immune surveillance. PD-1 inhibitors exert anti-tumor effect through blocking the binding of PD-1 to PD-L1, thereby reactivating T cells and immune system to attack cancer cells (16). The KEYNOTE-048 study has demonstrated that immune checkpoint inhibitors (ICI) therapy can be employed as a first-line treatment for locally advanced or metastatic HNSCC, and that the ICI+NAC regimen is more efficacious in enhancing OS in the overall patient population compared to chemotherapy in combination with cetuximab (53% vs 41%) (11). Recent study also demonstrates that the TP (paclitaxel+ cisplatin) plus ICI neoadjuvant treatment approach is superior to the cetuximab combined with TPF neoadjuvant treatment approach (82.5% vs 69.3%) (14).

Previous study indicates that patients with hypopharyngeal cancer or oropharyngeal cancer who received neoadjuvant chemotherapy combined with pembrolizumab before surgery possess 19% postoperative PCR rate, and the laryngeal preservation rate in patients with hypopharyngeal cancer reached 86.6% (13). In LAHPC who underwent surgical resection after ICI combined with NAC, the postoperative PCR rate reached 50% (10/20), and the overall laryngeal preservation rate was 95% (17). This neoadjuvant chemotherapy immunotherapy approach (camrelizumab plus nab-paclitaxel and cisplatin) has shown potential efficacy and acceptable safety in patients with resectable locally advanced squamous cell carcinoma of the head and neck (18). Previous studies have evaluated the use of PD-1 inhibitors as monotherapy in the neoadjuvant setting for patients with HNSCC, but the pathological response rates after surgery have been suboptimal (19). Thus, the PCR rate of ICI combined with NAC is significantly higher than that of single immunotherapy. In our study, the postoperative pathological PCR rate of the primary lesion reached 33.3%, and the laryngeal preservation rate reached 92.9%. In addition, we found that a total of 34 patients achieved pathological complete remission or partial remission, and the overall efficacy of ICI combined with NAC was 81%. When combined with PD-1 inhibitors, chemotherapeutic agents induce the release of tumor-associated antigens while promoting tumor cell death, thereby stimulating an antitumor immune response of activating T cells (20). Possibly, the high PCR after the treatment of ICI combined with NAC may be associated with reactivating T cell activity.

In our study, the PCR rate was 33.3% for primary lesions and 35.7% for metastatic cervical lymph nodes when PD-1 inhibitors were combined with NAC, with no statistically significant difference between the two groups. Notably, 9 patients (21.4%) achieved PCR in both the primary lesion and metastatic cervical lymph nodes. Some studies have reported that, compared with primary lesions, metastatic cervical lymph nodes exhibit a more pronounced therapeutic response to ICIs (21). However, the response rate to ICI and NAC in primary lesion was superior to that of the metastatic cervical lymph nodes (17). These discrepant findings warrant further investigation into tumor responsiveness to immunotherapy to better predict tumor response to ICIs.

Although the establishment of combined positive score (CPS) as a predictive biomarker for the efficacy of immune checkpoint blockade in HNSCC, there exists some ambiguity regarding the correlation between PD-1 expression and response to neoadjuvant therapy. According to the results of the KEYNOTE-012 study, higher CPS of PD-L1 is associated with a more significant treatment effect of ICI (22). The KEYNOTE-048 study demonstrates the improved survival outcomes in the patient population with CPS scores ranging from 1 to 20 (11). Patients with CPS >5 had greater clinical benefit from immunotherapy (13). In contrast, other study indicates no correlation between CPS and PCR (17). Our study demonstrated that CPS levels were also not associated with ICI treatment outcomes. Notably, we observed two patients with CPS <1 who achieved pCR following surgery. Therefore, a subset of patients with CPS <1 may still derive clinical benefit from ICI therapy. These observations may be attributed to various factors, including the complex interplay within the tumor microenvironment, the patient’s individual immune status, and the immunogenicity of the tumor itself. Thus, it is important to consider multiple factors when evaluating a patient’s response to ICI therapy, rather than relying solely on PD-L1 expression or CPS level (22). In previous studies, various molecular markers have good predictive value, including tumor mutation burden (TMB), microsatellite instability-high/deficiency of mismatch repair, and the proportion of CD8^+^ lymphocytes. However, challenges persist in assessing the efficacy of PD-1 inhibitors in combination with neoadjuvant chemotherapy (NAC) (11, 23). In the future, we need to further investigate how these markers can more accurately predict the efficacy of immunotherapy to guide clinical practice and the development of personalized treatment strategies.

In this study, NBI demonstrated a sensitivity of 57.69% and a specificity of 93.75% for identifying lesions after ICIs with NAC treatment. These findings are comparable to the diagnostic specificity of NBI for head and neck squamous cell carcinoma (93.2%) reported by Ni et al (15). NBI enhances the detection of squamous cell carcinoma in the oropharynx and hypopharynx compared to white-light endoscopy (24). Furthermore, we found that 73.53% of patients who achieved PCR or MPR of the primary lesion had a decreased NBI classification after ICI and NAC, while 87.5% of IPR patients still had a V type. It can be seen that NBI laryngoscope assessment of patients after ICI combined with neoadjuvant chemotherapy also has certain advantages, and it may be an important factor in predicting postoperative pathological results.

None of the patients in our study experienced immune-related severe grade III-V adverse events following the administration of ICI combined with NAC, and disease progression was not observed in any of the patients. Notably, after neoadjuvant therapy, one patient who underwent hemilaryngectomy experienced delayed wound healing postoperatively, and one patient who underwent total laryngectomy developed an infection around the tracheostoma. However, both patients recovered with conservative treatment, and their postoperative radiotherapy was not affected. Currently, there is no evidence to suggest that the combination of radiotherapy and ICI therapy improves survival rates (25, 26). Therefore, we did not administer ICI concurrently with postoperative radiotherapy or chemoradiotherapy.

However, our study has several limitations that should be acknowledged. First, this study includes a small sample size. Second, a single-center study with a limited sample size limits the generalizability of our findings and the ability to conduct more precise analyses of the correlation between NAC and prognosis. We will conduct a larger-scale multicenter clinical study to enhance the robustness of our results. Thirdly, the follow-up time is relatively short, and the long-term efficacy of NAC remains to be evaluated. Third, this is a retrospective, single-arm trial study, which may introduce selection bias and limit the level of evidence. Finally, our evaluation of the therapeutic effect is somewhat limited, and future studies should include more diverse outcome measures, such as speech function, swallowing function, and quality of life.

## 5 Conclusion

Combination therapy with PD-1 inhibitors and NAC has demonstrated superior efficacy and safety as a preoperative treatment for locally advanced hypopharyngeal and oropharyngeal cancer. Notably, this treatment regimen does not increase the risk of severe postoperative complications and has shown promising results in improving laryngeal preservation rates. However, further research and clinical trials are warranted to validate our findings and explore potential avenues for improvement to optimize treatment outcomes.

## Data Availability

The original contributions presented in the study are included in the article/supplementary material. Further inquiries can be directed to the corresponding authors.
